# Spatial–temporal-demographic and virological changes of hand, foot and mouth disease incidence after vaccination in a vulnerable region of China

**DOI:** 10.1186/s12889-022-13860-z

**Published:** 2022-08-01

**Authors:** Li Huang, Ting Wang, Xuxiang Liu, Yuansheng Fu, Sichen Zhang, Qinshu Chu, Tingyue Nie, Houmian Tu, Jian Cheng, Yinguang Fan

**Affiliations:** 1 Faculty of Clinical Medicine, Anhui Medical College, Hefei, 230601 Anhui China; 2 Department of Epidemiology and Health Statistics, School of Public Health, Anhui Medical University, Hefei, 230032 Anhui China; 3 Hefei Center for Disease Control and Prevention, Hefei, 230061 Anhui China

**Keywords:** Hand foot and mouth disease, Vaccine, Epidemiology, Enterovirus 71

## Abstract

**Background:**

The enterovirus 71 (EV-A71) vaccine has been used in Hefei for several years, and the epidemiological significance of vaccination in this area is unclear. We aims to explore the spatial–temporal-demographic and virological changes of hand, foot and mouth disease (HFMD) after vaccination in China.

**Methods:**

The data for HFMD from 2012 to 2020 were downloaded with the help of HFMD reporting system of Hefei Center for Disease Control and Prevention and combined with the EV-A71 vaccination status in Hefei. The study defined the period between 2012 to 2016 as the pre-vaccination period and explored the effect of vaccination on the incidence of HFMD by comparing the changes of HFMD before and after vaccination in terms of spatial, temporal, demographic and virological aspects.

**Results:**

During the study period, a higher incidence occurred in urban area and the random distribution changed to a slight cluster after vaccination. HFMD incidence had inconsistent seasonality over years, with one or two incidence peaks in varying years. The morbidity decreased from 215.22/105 in 2012–2016 to 179.81/105 in 2017–2020 (*p* < 0.001). Boys, 0–4 years old children and Scattered children were more susceptible to HFMD compared with the others, the proportions decreased after vaccination except in Scattered children. The main pathogenic enterovirus gradually changed from EV-A71 to Other Enteroviruses, especially coxsackieviruses A6 (CV-A6) after the implementation of EV-A71 vaccination.

**Conclusions:**

The EV-A71 vaccine was effective in reducing the incidence of HFMD and changing the spatial, temporal, demographic, and virological characteristic. These changes should be considered during the vaccination implementation to further reduce the disease burden of HFMD.

**Supplementary Information:**

The online version contains supplementary material available at 10.1186/s12889-022-13860-z.

## Background

Hand, foot and mouth disease (HFMD) is a virological infectious disease spread by human enterovirus, which is transmitted via close contact, faces and fomites [1]. The patients are usually children under 5 years of age [2], and usually present to healthcare facilities with fever, sore throat, and vesicular eruptions on their hands, feet, oral mucosa [3]. Severe cases are often accompanied by central nervous system symptoms or even severe respiratory symptoms [4], which could cause death.

HFMD tends to be a global public health issue and outbreaks have been reported in many parts of Asia since 1967 when it first appeared in Japan [5]. It appeared in China in 1981, the Chinese Center for Disease Control and Prevention has supervised 7 200 092 probable cases of HFMD, 2 457 of 82 486 patients with severe disease died (fatality rate 3.0%), and 1 617 of 1 737 laboratory confirmed deaths (93%) were associated with enterovirus 71 (EV-A71) from 2008 to 2012 [6]. HFMD has been listed as a Class C infectious disease in China since 2008, which must be reported with 24 h [7]. HFMD morbidity has been in the top three in Class C infectious diseases for the past five years [8–12].

EV-A71 is a member of the genus Enterovirus within the family Picornaviridae which is one of the most important neurotropic viruses causing HFMD, and it has posed a serious threat to public health worldwide [13]. Numerous Member States in the Western Pacific Region have experienced large HFMD epidemics associated with EV-A71 infection [4, 14]. For example, the investigators in Japan suggested that bimodal seasonal peaks in HFMD epidemics were attributable to EV-A71 epidemics [15]. Enteroviral cultured from a large epidemic of HFMD in Singapore proved that, EV-A71 was the most frequently isolated virus from both HFMD patients and non-HFMD patients [16]. EV-A71 was predominant in laboratory-confirmed cases, accounting 45% of mild, 80% of severe, and 93% of fatal cases in China from 2008 to 2012 [6], and caused 52.78% HFMD cases and 86.36% severe cases in Anhui Province before the inactivated EV-A71 vaccine was implemented [17], suggests that controlling the EV-A71 infection plays a vital role in preventing HFMD. Furthermore, another type of enterovirus, coxsackieviruses A6 (CV-A6), has been increasingly associated with HFMD cases or outbreaks globally over the past decade [18].

Since most people show asymptomatic infection after human enteroviruses infection, it is difficult to be detected clinically, so it is difficult to carry out effective isolation measures, which may easily lead to further spread of the disease [3]. Therefore, vaccination of susceptible people may be an effective method for preventing HFMD epidemics. Despite EV-A71 vaccine being available to people for a fee since August 2016 in the region of our study, the real-world impact of the vaccination program remains unknown. We therefore aimed to provide scientific evidence for HFMD control and prevention by analyzing the disease data from 2012 to 2020, and to explore the effect of EV-A71 vaccine in Hefei, China.

## Methods

### Data source

Hefei, which is 11 445.1 km^2^ in area, is the capital of Anhui Province and an important city in the Yangtze River Delta. Hefei is a metropolis which has 9 369 881 permanent residents (15.35% in Anhui) in 2020 [19]. The center of Hefei consists of 4 districts with a high population density surrounded by another 5 areas with a lower population density. Hefei has a semitropical climate with spring beginning in March.

The daily data of HFMD in Hefei from January 1st 2012 to December 31st 2020 was downloaded from the direct reporting system of the infectious disease network of China. The downloaded data covered all HFMD cases since all cases must be uploaded through the system, the download data covered all cases. A total of 143 380 records were downloaded and included in the analysis. According to the classification standards of the reporting system, children in childcare centers or kindergartens were classified as Kindergarten children, and the rest as Scattered children. Demographic information was obtained from the statistical yearbook of Anhui Provincial Bureau of Statistics [20] and Hefei Municipal Bureau of Statistics [21].

### Laboratory analysis

According to the Hand, Foot and Mouth Disease Prevention and Control Guidelines, the sample size, using random sampling, was more than 300 cases per year, and was expanded to 500 cases per year since 2019 [22]. Before 2018, the test standard for HFMD in the Laboratory Department of Hefei Center for Disease Control and Prevention was to divide the positive results into three categories: coxsackieviruses A16 (CV-A16), EV-A71 and Other Enteroviruses. Since 2018, the laboratory reported additional results of CV-A6 and coxsackieviruses A10 (CV-A10), which belong to Other Enteroviruses.

### Statistical analysis

To explore the characteristics of HFMD from the perspectives of space, time, demography and virology in Hefei from 2012 to 2020. The spatial distribution of HFMD incidence was analyzed by using the colored map according to the administrative division of Hefei City. The periodicity and seasonality of HFMD were discussed in daily number of cases and monthly morbidity. In calculating the *Moran’s I* index, the conceptualization was chosen as CONTIGUITY EDGES CORNERS, which compute surface elements that share boundaries, nodes, or overlaps with the target element. Specifically, the EUCLIDEAN method was used for calculating the distance between each element and its neighbors. We aimed to identify the high-risk groups of HFMD through the analysis of age, sex and childcare patterns. For the age stratification analysis, the HFMD cases were divided into 4 ages groups: 0–4 years old, 5–9 years old, 10–14 years old, and ≥ 15 years old. In the subsequent analysis, the group aged 0–4 years old was divided into 5 groups to determine the high-risk age. The laboratory reported three categories of HFMD pathogens: CV-A16, EV-A71 and Other Enteroviruses, but two representative types of Other Enteroviruses, CV-A6 and CV-A10, were reported separately since 2018. We combined 2012–2017 as pre-vaccination and 2018–2020 as post-vaccination periods.

Statistical analysis was done using R 4.0.5 and ArcGIS 10.8 software. The incidence was calculated as the number of cases divided by the number of permanent residents at the end of the year (cases per 10^5^ people). Vaccination coverage rates were calculated as the number of vaccinations divided by the number of people under five years of age (vaccination per 10^4^ children < 5 years). The Newborn-vaccination rate was calculated as the number of completed vaccinations divided by the number of births in the year (per 100). The Chi-squared test was used for analyzing categorical data and the level of significance for all analysis was *p* < 0.05.

## Results

### Spatial characteristics

**Fig. 1:**
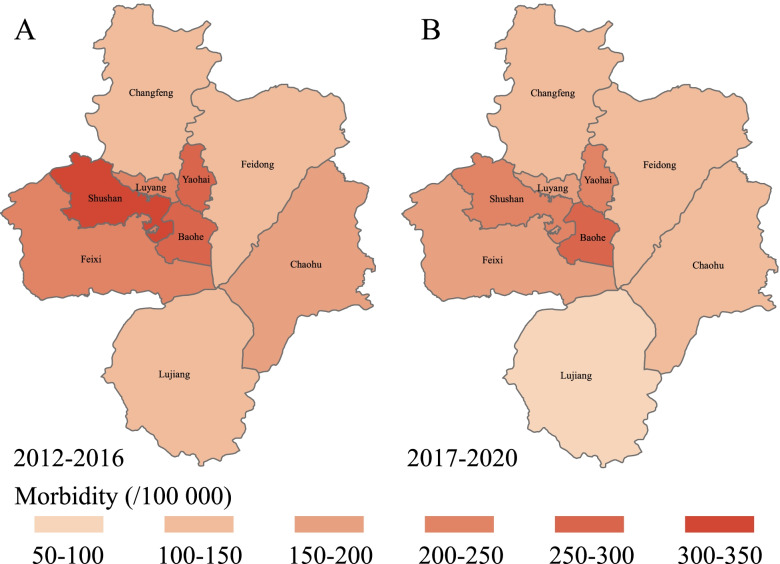
Regional distribution of HFMD before and after vaccination in Hefei from 2012 to 2020

The total number of HFMD cases from 2012 to 2020 was 143 380. The number of HFMD cases varied from 8 511 (5.94%) cases in Lujiang County to 31 836 (22.20%) cases in Shushan District (Table S1). The 4 districts located in the center had higher incidence than the other areas, and as shown in the maps (Fig. 1, Fig. S1), the relative farther away from the city center the lower the incidence. However, Lujiang County was an exception which had the lowest incidence, the number of cases increased to 2 385 with an incidence of 244.62/10^5^ in 2014, making it the fourth highest area with HFMD cases. Spatial autocorrelation analysis (Table 1) showed that the distribution of HFMD among 9 districts in Hefei City was random from 2012 to 2020, except for 2017 (*Moran’s I* = 0.197, *p* = 0.077) and 2018 (*Moran’s I* = 0.258, *p* = 0.037). After vaccination, the random distribution of HFMD changed to a slight cluster among Districts (*Moran’s I* = 0.103, *p* = 0.215 *vs*. *Moran’s I* = 0.268, *p* = 0.051).

**Table 1:** Spatial and temporal autocorrelation analysis of HFMD in Hefei from 2012 to 2020

Year	*Moran’s I*	*Z* score	*p* value	Distribution
2012	0.096	1.331	0.183	Random
2013	-0.045	0.440	0.661	Random
2014	0.014	0.762	0.446	Random
2015	0.139	1.469	0.142	Random
2016	0.124	1.386	0.166	Random
2017	0.197	0.033	0.077	Clustered
2018	0.259	2.091	0.037	Clustered
2019	-0.039	0.472	0.637	Random
2020	0.037	0.991	0.322	Random
2012–2016	0.103	1.240	0.215	Random
2017–2020	0.268	1.953	0.051	Clustered
Count	0.156	1.541	0.123	Random

### Temporal characteristics

**Fig. 2:**
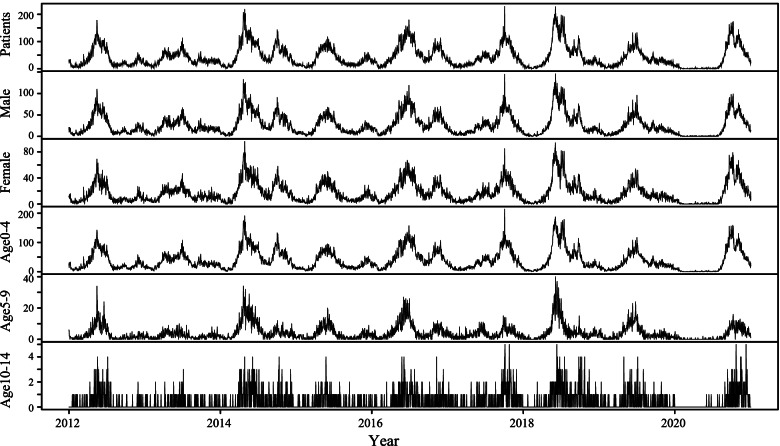
Temporal changes of HFMD at daily level in Hefei from 2012 to 2020

From 2012 to 2020, the total number of patients and average incidence were 143 380 and 198.73/10^5^ respectively. The monthly number of cases ranged from 4 620 in June 2018 to 16 in February 2020. Two seasonal epidemic patterns are evident (Fig. 2). There was an incidence peak in spring from April to July in 2012, 2013, 2015 and 2019, while there was an autumn peak from September to November in 2017 and 2020. Unlike the aforementioned unimodal patterns, the seasonality of 2014, 2016 and 2018 manifested as a bimodal pattern with a higher peak in spring and a lower peak in autumn (Fig. S2).

After EV-A71 vaccine implementation, the seasonality and periodicity of HFMD incidence became atypical, with no epidemic peak noted for the first time in the spring of 2017. The incidence was extremely low in the first half of 2020, with the cumulative number of cases in the first seven months accounting for only 3.59% (437/12 184) of the whole year.

There were 114 severe cases ie 11, 22, 70, 3, 2, 3, 3, 0, 0 cases per year from 2012 to 2020 (Table S1). The highest incidence of severe HFMD was in 2014, reaching 70 (61.40% of 114). Thereafter, the incidence of severe cases was controlled at a lower level and no severe cases were reported in 2019 and 2020. Three children died of severe HFMD in 2013 and 2014, about three days after they were diagnosed.

### Demographic characteristics

The HFMD morbidity decreased (Table 2) from 215.22/10^5^ in 2012–2016 to 179.81/10^5^ in 2017–2020 (*p* < 0.001) following vaccination. Of the total 143 380 cases, 60.65% were males and 39.35% were females. The morbidity decreased in both genders after vaccination (*p* < 0.001) including a decreased ratio of male to female (158.77 *vs*. 147.98, *p* < 0.001).

**Table 2:** Changes in demographic characteristics before and after vaccination

Variables	2012–2016	2017–2020	*p* value
Total	82 944(215.22)	60 436(179.81)	< 0.001
Sex [n (/10^5^)]			
Male	50 891(262.24)	36 065(209.94)	< 0.001
Female	32 053(167.53)	24 371(148.31)	< 0.001
Age group (years old) [n (/10^5^)]			
0–4	74 200(3 088.63)	53 744(2 635.19)	< 0.001
5–9	7 709(308.20)	5 557(262.24)	< 0.001
10–14	715(32.69)	716(36.85)	0.312
≥ 15	320(1.02)	419(1.52)	0.013
Childcare patterns [n (%)]			
Scattered children	56 245(67.81)	42 702(70.66)	< 0.001
Kindergarten children	23 708(28.58)	14 784(24.46)	
Student	2 762(3.33)	2 637(4.36)	
Other patterns	229(0.28)	313(0.52)	
Residence [n (/10^5^)]			
Yaohai	13 652(286.60)	9 803(226.74)	< 0.001
Luyang	6 796(211.47)	4 396(160.29)	< 0.001
Shushan	18 280(300.78)	13 556(236.76)	< 0.001
Baohe	13 251(294.77)	10 320(250.35)	< 0.001
Chaohu	7 443(190.80)	4 185(134.19)	< 0.001
Changfeng	4 418(139.33)	4 118(147.85)	0.203
Feidong	6 277(145.50)	4 781(133.79)	0.039
Feixi	7 714(206.49)	5 879(177.69)	< 0.001
Lujiang	5 113(105.06)	3 398(86.88)	< 0.001
Pathogen [n (%)]			
CV-A16	506(29.68)	426(24.18)	0.100
EV-A71	603(35.37)	80(4.54)	< 0.001
Other Enteroviruses^a^	596(34.96)	885(50.23)	< 0.001
CV-A6	-	357(20.26)	-
CV-A10	-	14(0.79)	-
Severe cases [n (%)]	108(0.13)	6(0.01)	< 0.001

^a^Other Enteroviruses included CV-A6 and CV-A10 prior to 2018, both were not included after 2018

**Fig. 3:**
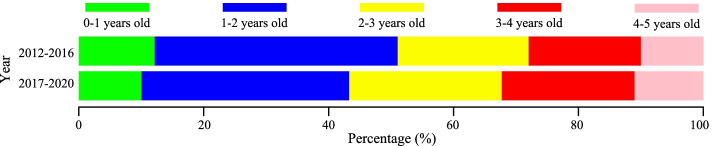
Age proportion of the group aged 0–4 years old with HFMD cases before and after vaccination in Hefei from 2012 to 2020

A total of 127 944 cases were under the age of 5 (89.23%), the incidence changed from 3 088.63/10^5^ to 2 635.19/10^5^ in this group after vaccination (*p* < 0.001). There was a slight increase from 32.69/10^5^ to 36.85/10^5^ (Table 2, Fig. S3) in the 10 to 14-year age group after vaccination (*p* = 0.312). The proportion of patients with HFMD under 2 years old was decreased (Fig. 3).

Scattered children (98 947 cases, 69.01%) were more than Kindergarten children (38 492 cases, 26.85%) or student (5 399 cases, 3.77%) among the HFMD cases. Scattered children accounted for 76.60% (98 001/127 944) among the 0–4 years old children with HFMD. The proportions of childcare patterns have changed (*p* < 0.001), showing an increase in the proportion of Scattered children (67.81% *vs*. 70.66%). Among the 114 severe cases, 74 (64.91%) were males, 101 (88.60%) were children under 5 years old, and 92 (80.70%) were Scattered children. The proportion of severe cases dropped sharply after vaccination (*p* < 0.001).

### Virological characteristics

**Fig. 4:**
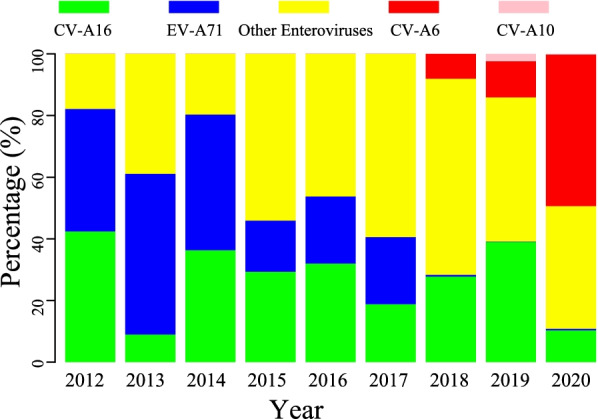
Enteroviruses proportion of HFMD in Hefei from 2012 to 2020

The frequency of EV-A71 cases declined in 2014 and 2017 and since then, the incidence of EV-A71 has remained at a very low level. The incidence of Other Enteroviruses has relatively increased, of which, CV-A6 was more prominent (Fig. 4). Since 2017, there was a sharp decline of EV-A71 cases from 134 (39.76%) to 3 (0.55%) in 2020 (*p* < 0.001) and a noticeable increase of CV-A6 cases of 27 (8.06%) in 2017 to 266 (49.17%) in 2020 (*p* < 0.001). The proportion of CV-A16 has dropped form 29.68% in 2017 to 24.18% in 2020 (*p* = 0.100). However, there was an increase in Other Enteroviruses (34.96% *vs*. 50.23%, *p* < 0. 001).

There was a difference of dominant enterovirus type in different seasons (Fig. S4). In general, the reported three main enterovirus types accounted for an equal proportion in the spring peak, while the proportion of Other Enteroviruses stood out in the autumn peak (Fig. S5). The proportion of the reported three main types was not balanced in spring and summer before vaccination since EV-A71 was dominant. However, after vaccination, the proportion of EV-A71 accounted for less cases, but the proportion of Other Enteroviruses changed significantly to made up a large part in autumn (Fig. S6).

### The EV-A71 vaccine coverage rates

**Fig. 5:**
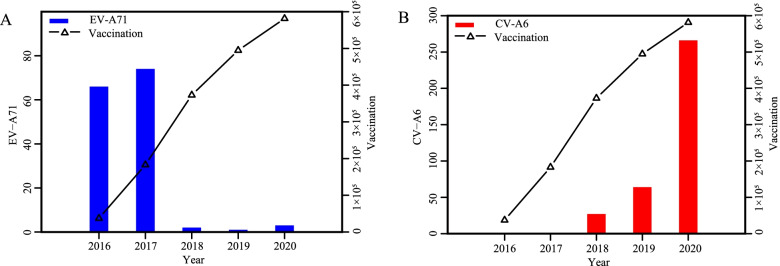
Trend in cumulative EV-A71 vaccination compared to EV-A71 or CV-A6 positivity in Hefei from 2016 to 2020

From 2016 to 2020, the EV-A71 total vaccination rate in Hefei increased from 7.64/10^4^ in 2016 to 29.93/10^4^ in 2017 and 39.09/10^4^ in 2018 but decreased from 25.59/10^4^ in 2019 to 14.68/10^4^ in 2020 (Table 3). The completion-vaccination rate increased from 0.14/10^4^ in 2016 to 0.84/10^4^ in 2017 and 1.12/10^4^ in 2018 but decreased from 0.74/10^4^ in 2019 to 0.45/10^4^ in 2020. The newborn-completion vaccination rates increased from 9.54% in 2016 to 45.46% in 2017 and 71.33% in 2018 but also decreased from 58.53% in 2019 to 53.05% in 2020. The completion rates of the two injections were 30.38%, 45.83%, 47.74%, 49.83%, 48.50% per year from 2016 to 2020 respectively. Since the implementation of the EV-A71 vaccination, the vaccination rates in Hefei have shown a trend of rising in 2016 and then decreasing in 2020, with the highest rate in 2018. Following the EV-A71 vaccination implementation, the incidence of EV-A71 decreased while Other Enteroviruses especially CV-A6 increased (Fig. 5, Fig. S7).

**Table 3:** EV-A71 vaccination rates in Hefei from 2016 to 2020

Vaccination rates	2016	2017	2018	2019	2020	Cumulative
Total-vaccination rates (/10^4^)	7.64	29.93	39.09	25.59	14.68	98.38
Completion-vaccination rates (/10^4^)	0.14	0.84	1.12	0.74	0.45	48.50
Newborn-completion-vaccination rates (%)	9.54	45.46	71.33	58.53	53.05	47.17
Completion rates (%)	30.38	45.83	47.74	49.83	48.50	46.70
0–4 years old vaccination rates (%)	7.64	29.93	39.09	25.59	14.68	98.38

## Discussions

We found a higher incidence in densely populated urben areas which changed to a slight cluster after vaccination. There was a significant decrease in HFMD morbidity during the pre-vaccination period 2012 to 2016 (215.22/10^5^) compared to the post vaccination period 2017 to 2020 (179.81/10^5^). Boys, children under 5 years and Scattered children were more likely to develop HFMD compared with other individuals; the proportions decreased after vaccination except for Scattered children. The incidence of the commonest pathogenic enterovirus (EV-A71), causing HFMD in Hefei was significantly reduced after EV-A7 vaccination with a further significant reduction in severe HFMD. There was an increase in Other Enteroviruses causing HFMD, especially CV-A6.

We have shown that the four districts located in the center of Hefei had higher incidence than the other areas, and the relative distance from the city center the lower the incidence. After vaccination, the random distribution of HFMD changed to slight cluster among Districts. The regional distribution of HFMD in Sichuan Province also conforms to this feature [23]. It may be the higher population density and mobility in the four districts enabled easier for enterovirus to spread [24, 25], which indicates that prevention and control of transmission routes should be a focus of attention in populated areas.

However, in remote rural area or economically underdeveloped areas, the health awareness of people may not be high enough resulting in patients with HFMD not seeking the health service, as indicated by studies which showed a negative correlation between socioeconomic development and the incidence of severe illness suggesting that cases in less economically developed areas were more likely to develop severe illness [25, 26].

Lujiang County usually had the lowest incidence but the morbidity escalated in 2014. This could be due to previous decreased health vigilance in this area andpossibly people with low antibody levels making it difficult to respond to outbreak of HFMD [27]. Furthermore, following vaccination, the areas of higher incidence were usually the center of Hefei, and the random distribution of HFMD changed to slight cluster among districts, suggesting that the center of Hefei or urban are the key areas for prevention and control of HFMD.

According to previous studies [6], HFMD in northern China presents a single-peak pattern in spring and summer, and a double-peak pattern in southern China, while both patterns can occur in Hefei as shown by an autumn peak from September to November in 2017 and 2020. This may be due to the position of Hefei being in middle of China resulting in a semitropical climate. The HFMD epidemic in Guangxi Province had the same autumn peak in 2017 after vaccination [28]. The extremely low incidence in Hefei from February to July 2020 may be due to the COVID-19 outbreak in China in late 2019 [29]. Hefei suspended school and work during the period, strengthened respiratory transmission prevention and control efforts, effectively reduced human-to-human transmission and respiratory transmission [30], which resulted in respiratory precautions playing an effective role in HFMD prevention and control.

After EV-A71 vaccination, the seasonality and periodicity of HFMD incidence became atypical, with no epidemic peak being noted for the first time in the spring of 2017. A study using the Distributed Lag Nonlinear Model found that HFMD incidence in children had a nonlinear relationship with the daily mean temperature, and there was a significant lag effect in Nanjing, China [31], suggesting that HFMD seasonality was related to climate, which also showed in our study. The seasonal HFMD patterns has implications for seasonal and periodic HFMD prevention programs. Therefore, in combination with the cyclical nature of the virus transmission, the analysis of the HFMD temporal characteristics before and after vaccination may be helpful to anticipate the development of epidemics timeously. However, the impact of socioeconomic factors and other health conditions on the temporal characteristics of HFMD requires further analysis.

Children under 5 years especially males had a higher proportion of HFMD in Hefei, which was consistent with other areas [31–33]. The morbidity decreased in in males and females following vaccination, with males remaining at higher risk of HFMD, which were consistent with studies in Guangxi and Zibo cities of China [28, 34]. There was a slight decrease in the group aged 0–4 years old, especially in children aged less than 1 year and aged 1 year old. Meanwhile, boys and kids under 1 year old were more vulnerable to temperature variations [31], suggests that more effectual protection are needed to be adopted for the key groups of HFMD.

Scattered children were much more affected by HFMD compared to Kindergarten children and students, with HFMD increasing in Scattered children after EV-A71 vaccination. These changes were consistent with Guangxi [28], and the EV-A71 vaccine reduced 42.9% of HFMD cases among Scattered children in Guangdong [35]. This may be due to the large proportion of Scattered children under 5-year age group, and it is more difficult to implement primary, secondary and tertiary prevention among these children.

Although the decreased incidence of severe HFMD may be mainly attributed to the improvement in health conditions, the incidence of severe HFMD decreased as the incidence of EV-A71 also dropped, demonstrating the clinical significance of prevention and control of EV-A71 following vaccination. It was also consistent with other cities in China where have implemented EV-A71 vaccination, for example, the proportion of EV-A71 among severe cases dropped from 37.9% to 14.1% following vaccination in Nanchang [36].

The main cause of HFMD used to be EV-A71 but showed a significant decline in 2015 and has been controlled at a very low level since 2018. The 2015 decline may be related to the improvement of health conditions and the increased awareness of infection prevention and control in of the community following an HFMD epidemic in 2014 [37, 38]. The decline in 2018 may be related to the gradual increased rate of EV-A71 vaccination, which began in the second half of 2016 [32] and to the higher level of EV-A71 serum neutralizing antibody in healthy people [39].

We have shown an upward trend in EV-A71 vaccination rates and a downward trend in EV-A71 causing HFMD. Furthermore, the reported three main enterovirus types accounted for a balanced proportion during the spring peak, while the proportion of Other Enteroviruses stood out in the autumn peak. However, following vaccination, the proportion of EV-A71 accounted for less HFMD cases, but the proportion of Other Enteroviruses changed significantly, contributing much more to autumn cases. These changes in seasonal patterns has been reported in Nanchang city of China that the proportion of CV-A6 was prominent at autumn following vaccination, and particularly among severe cases [36]. Furthermore, these findings have major implications for prevention of HFMD in different seasons, such as emphasizing EV-A71 vaccination in spring and summer while increasing protection against Other Enteroviruses in autumn and winter.

The EV-A71 vaccine has no cross-immunity against CV-A16 and Other Enteroviruses [39], thus increasing the proportion of Other Enteroviruses causing HFMD, especially the sharp increase in CV-A6 we have shown. The increasing prevalence of CV-A6 may result in future HFMD outbreaks, however it may be missed as a cause due to its complex clinical manifestations [40]. Furthermore, it was also shown that CV-A6 accounted for 62.33% of severe HFMD in Kunming in 2018 [32]. The prevention and control of HFMD should be diversified and the Other Enteroviruses should also be monitored. EV-A71 vaccination may also reduce morbidity and mortality in HFMD cases since these patients usually have a higher proportion of CNS diseases and increased mortality [41].

We have shown high newborn-completion-vaccination rates (71.33% in 2018) in Hefei while Guangzhou had a 15.11% rate in 2018 [42]. In Ningbo, the newborn-completion-vaccination rate was 44.23% [43], and the incidence density was as high as 5 945.50/10^5^ person-years [33]. Countries such as Japan and Singapore also have a high prevalence of HFMD, but did not use the EV-A71 vaccine, although EV-A71 prevalence dropped in 2015 but subsequently increased thereafter [44, 45]. The EV-A71 vaccine implementation is indeed important, but the cyclical nature of the virus transmission should also be considered during implementation.

Since the implementation of EV-A71 vaccination in Hefei, the overall vaccination rates have shown a trend of initially rising and then decreasing, with the highest rate in 2018. However, the completion rates of 2-dose vaccination were not high, with a cumulative rate of 46.70%, which will reduce the overall effectiveness of the vaccine. Therefore, future attention should be focused to continuously improve maintenance of vaccination. Notably, the guidelines recommend completion of EV-A71 with two doses before 12 months of age suggesting timeliness was considered to be an indicator of the effectiveness of EV71 vaccine [43, 46].

Our study has several limitations. Firstly, we were unable to associate the seasonality of HFMD morbidity following vaccination since monthly vaccinations are not done. We thus used a combination of annual vaccinations and HFMD incidence to observe the effect of vaccination on the trend of HFMD which did not account for delayed effects. Secondly, we could not describe the HFMD morbidity in vaccinated people since individual vaccination details were not available, so we could only use the EV-A71 vaccination status of the whole population to make inferences. Therefore, we were unable to investigate the effects of EV-A71 vaccine on different enteroviruses directly. Thirdly, we are unable to analyze the difference between individual people who complete the two doses of vaccine schedule and those who did not complete it.

## Conclusions

The EV-A71 vaccine was effective in reducing the incidence of HFMD and changing its spatial, temporal, demographic, and virological characteristic. The prevention and control of HFMD should be focused in areas with high population density, on boys, children under 5 years old and Scattered children. There was an increase in Other Enteroviruses, especially CV-A6, which may require focused surveillance to anticipate future HFMD epidemics and exploration in formulating a multivalent HFMD vaccine. Further research needs investigation of Other Enteroviruses in circulation and antibody responses to the EV-A71 vaccine.

## Supplementary Information

**Additional file 1: Fig. S1 **Regional distribution of HFMD before and after vaccination in Hefei from 2012 to 2020.** Fig. S2** Temporal changes of HFMD morbidity at monthly level in Hefei from 2012 to 2020.** Fig. S3 **Age proportion of HFMD patients before and after vaccination in Hefei from 2012 to 2020.** Fig. S4 **Monthly enteroviruses proportion of HFMD in Hefei from 2012 to 2020.** Fig. S5** Monthly enteroviruses proportion of HFMD before vaccination in Hefei from 2012 to 2016.** Fig. S6 **Monthly enteroviruses proportion of HFMD after vaccination in Hefei from 2017 to 2020.** Fig. S7 **Trend in cumulative EV-A71 vaccination compared to Other Enteroviruses positivity in Hefei from 2016 to 2020.** Table S1 **Characteristics of HFMD in Hefei City from 2012 to 2020

## Data Availability

Data cannot be shared publicly because of the Infectious disease reporting system contains patient privacy and requires employee account login. Data are available from the Hefei Center for Disease Control and Prevention Institutional Data Access (E-mail: 526,893,544@qq.com) for researchers who meet the criteria for access to confidential data. The datasets analysed during the current study are available from the corresponding author on reasonable request.

## Abbreviations

HFMD: Hand, foot and mouth disease
EV-A71: Enterovirus 71
CV-A6: Coxsackieviruses A6
CV-A16: Coxsackieviruses A16
CV-A10: Coxsackieviruses A10
