# Correction: Spatial–temporal-demographic and virological changes of hand, foot and mouth disease incidence after vaccination in a vulnerable region of China

**DOI:** 10.1186/s12889-022-13941-z

**Published:** 2022-08-23

**Authors:** Li Huang, Ting Wang, Xuxiang Liu, Yuansheng Fu, Sichen Zhang, Qinshu Chu, Tingyue Nie, Houmian Tu, Jian Cheng, Yinguang Fan

**Affiliations:** 1 Faculty of Clinical Medicine, Anhui Medical College, Hefei, 230601 Anhui China; 2 Department of Epidemiology and Health Statistics, School of Public Health, Anhui Medical University, Hefei, 230032 Anhui China; 3 Hefei Center for Disease Control and Prevention, Hefei, 230061 Anhui China

**Correction: BMC Public Health 22, 1468 (2022)**

**https://doi.org/10.1186/s12889-022-13860-z**

The original publication of this article [1] contained an incorrect author name. The incorrect and correct name are shown below. The original article has been updated.

Author name

- The incorrect author name is: Jian Chen
- The correct author name is: Jian Cheng
