# Supplementary material for: Spatial–temporal-demographic and virological changes of hand, foot and mouth disease incidence after vaccination in a vulnerable region of China
Source: BMC Public Health. 2022 Aug 1;22:1468. doi: 10.1186/s12889-022-13860-z (PMC9342842; doi:10.1186/s12889-022-13860-z)
Supplement: Supplementary file 1 — Additional file 1: Fig. S1 Regional distribution of HFMD before and after vaccination in Hefei from 2012 to 2020. Fig. S2 Temporal changes of HFMD morbidity at monthly level in Hefei from 2012 to 2020. Fig. S3 Age proportion of HFMD patients before and after vaccination in Hefei from 2012 to 2020. Fig. S4 Monthly enteroviruses proportion of HFMD in Hefei from 2012 to 2020. Fig. S5 Monthly enteroviruses proportion of HFMD before vaccination in Hefei from 2012 to 2016. Fig. S6 Monthly enteroviruses proportion of HFMD after vaccination in Hefei from 2017 to 2020. Fig. S7 Trend in cumulative EV-A71 vaccination compared to Other Enteroviruses positivity in Hefei from 2016 to 2020. Table S1 Characteristics of HFMD in Hefei City from 2012 to 2020 [file 12889_2022_13860_MOESM1_ESM.docx]

**Supplementary Materials**


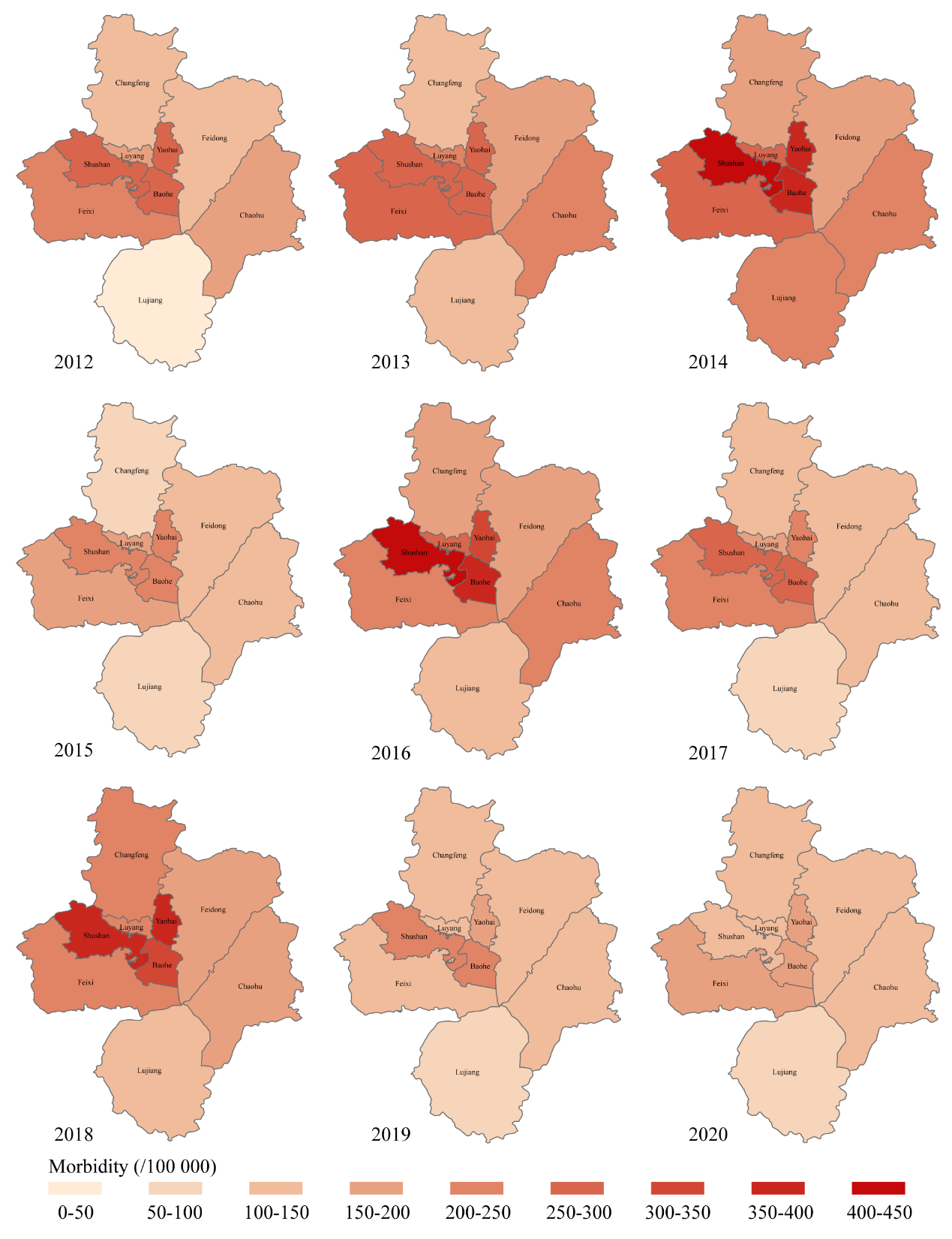


**Fig. S1** Regional distribution of HFMD before and after vaccination in Hefei from 2012 to 2020


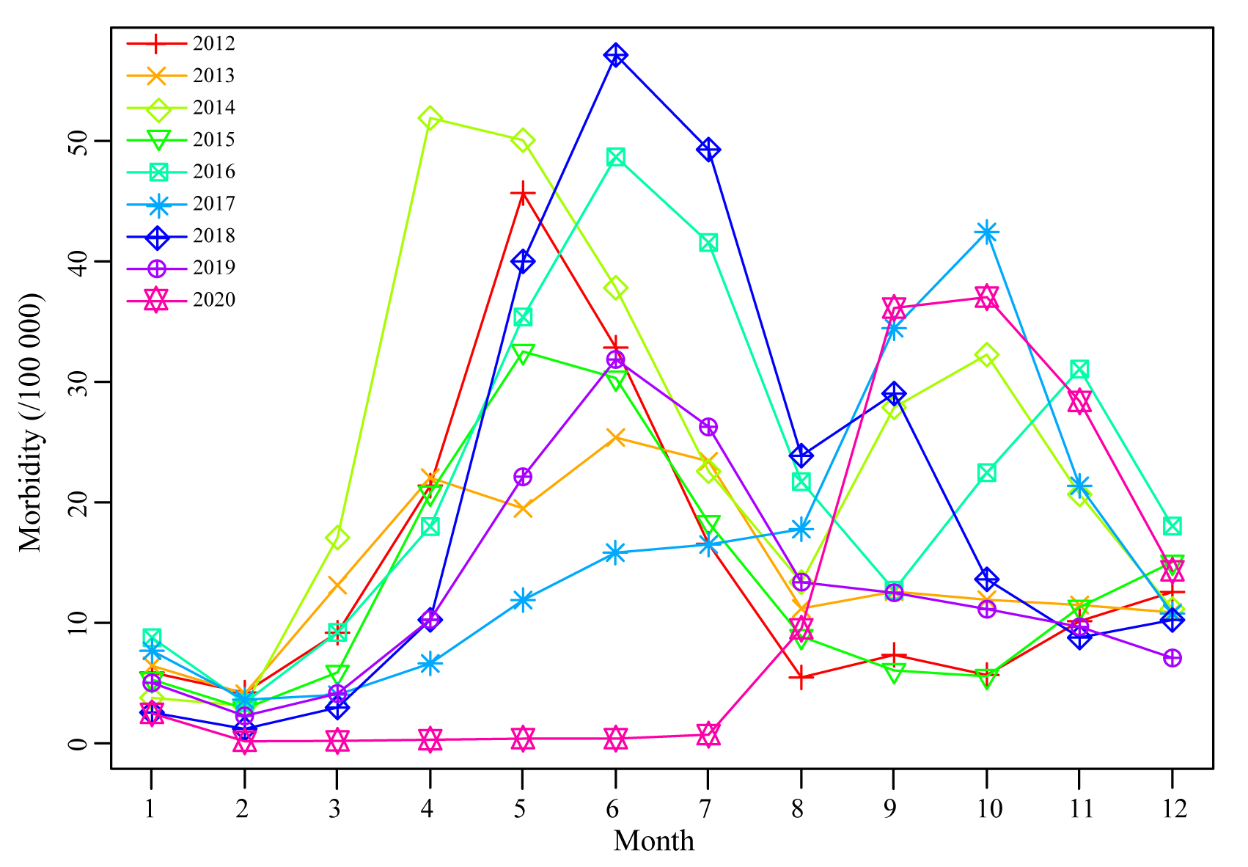


**Fig. S2** Temporal changes of HFMD morbidity at monthly level in Hefei from 2012 to 2020


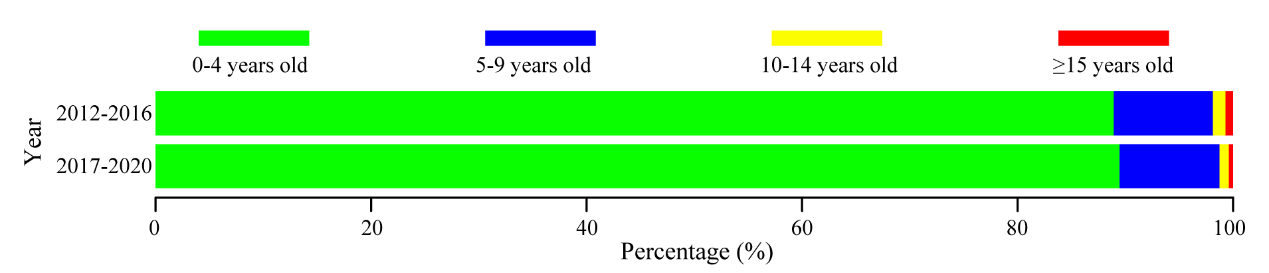


**Fig. S3** Age proportion of HFMD patients before and after vaccination in Hefei from 2012 to 2020


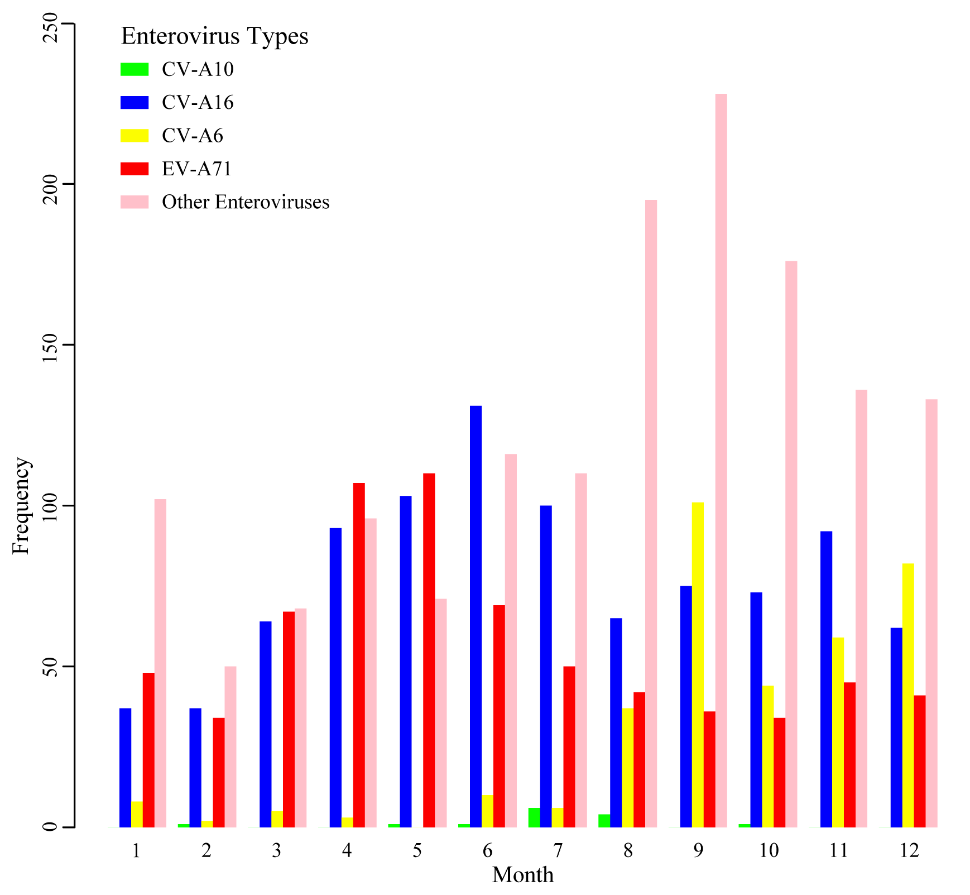


**Fig. S4** Monthly enteroviruses proportion of HFMD in Hefei from 2012 to 2020


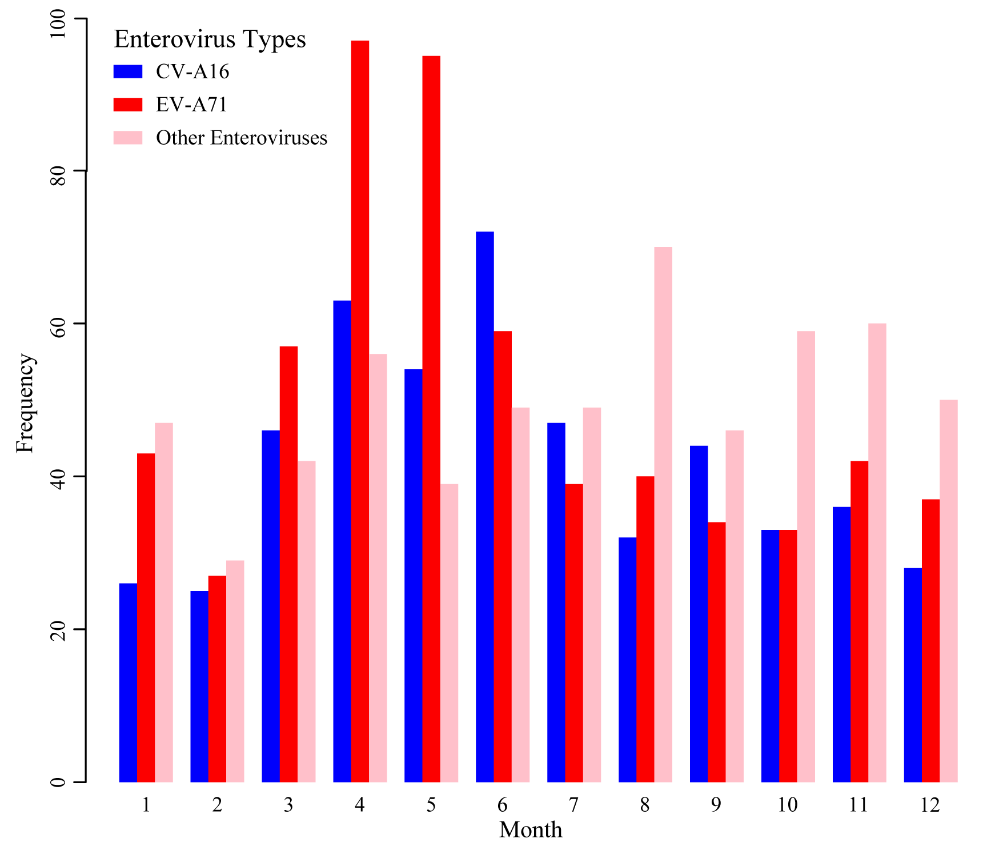


**Fig. S5** Monthly enteroviruses proportion of HFMD before vaccination in Hefei from 2012 to 2016


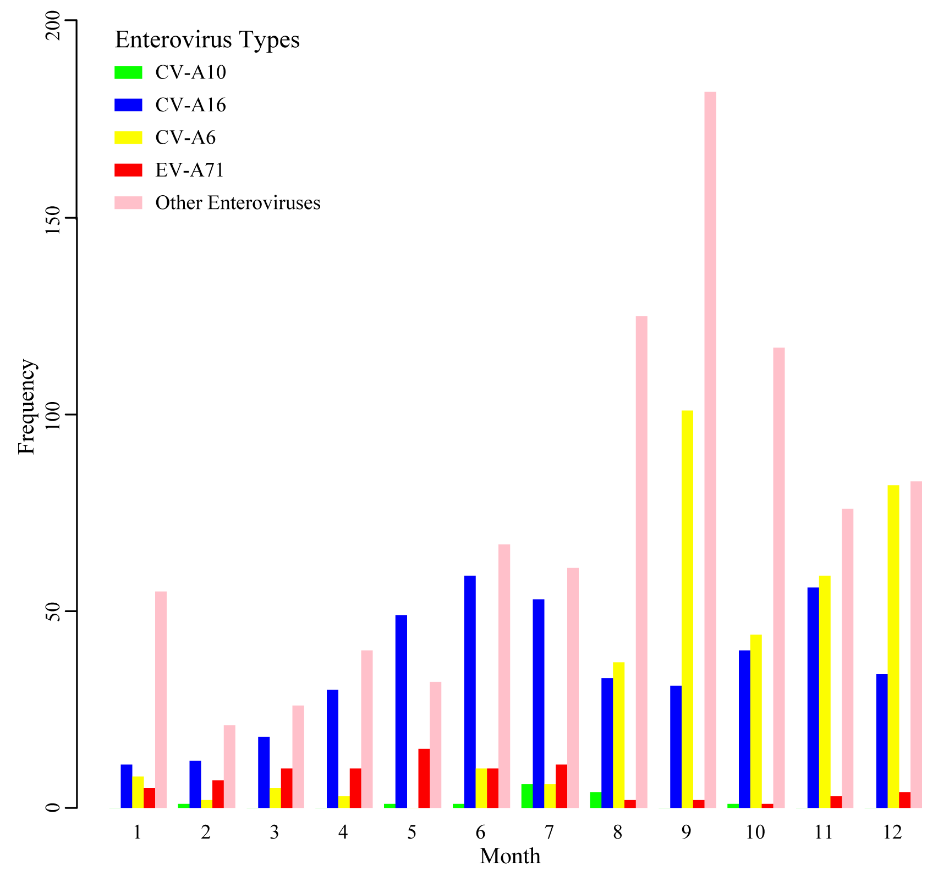


**Fig. S6** Monthly enteroviruses proportion of HFMD after vaccination in Hefei from 2017 to 2020


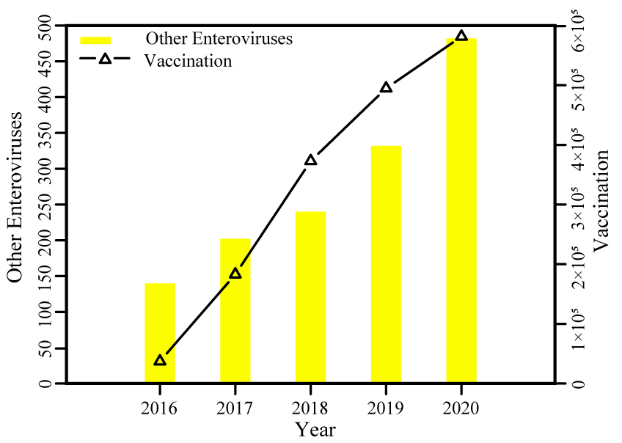


**Fig. S7** Trend in cumulative EV-A71 vaccination compared to Other Enteroviruses positivity in Hefei from 2016 to 2020

**Table S1** Characteristics of HFMD in Hefei City from 2012 to 2020

| **Variables** | **2012** | **2013** | **2014** | **2015** | **2016** | **2017** | **2018** | **2019** | **2020** | **total** |
| --- | --- | --- | --- | --- | --- | --- | --- | --- | --- | --- |
| Total [n (/10^5^)] | 13396(176.91) | 13096(172.06) | 22446(291.66) | 12683(162.81) | 21323(270.97) | 15370(192.97) | 20130(248.92) | 12752(155.72) | 12184(130.03) | 143380(198.73) |
| Sex [n (/10^5^)] |  |  |  |  |  |  |  |  |  |  |
| Male | 8444(223.49) | 8035(211.41) | 13608(347.76) | 7656(193.87) | 13148(331.60) | 9338(230.17) | 12165(294.20) | 7674(184.25) | 6888(142.85) | 86956 |
| Female | 4952(130.53) | 5061(132.81) | 8838(233.62) | 5027(130.88) | 8175(209.40) | 6032(154.35) | 7965(201.54) | 5078(126.19) | 5296(116.44) | 56424 |
| Age [n (/10^5^)] |  |  |  |  |  |  |  |  |  |  |
| 0-4 | 11970(2549.72) | 12107(2553.19) | 19830(4013.49) | 11296(2369.39) | 18997(3893.80) | 13831(2842.02) | 17780(3658.22) | 11090(2330.90) | 11043(1868.53) | 127944 |
| 5-9 | 1269(266.02) | 844(168.52) | 2301(452.32) | 1217(241.84) | 2078(406.27) | 1245(239.00) | 1988(372.46) | 1452(264.64) | 872(169.07) | 13266 |
| 10-14 | 128(6.55) | 83(14.32) | 208(24.61) | 116(12.29) | 180(15.57) | 194(21.10) | 245(23.19) | 138(13.76) | 139(31.51) | 1431 |
| ≥15 | 29(0.47) | 62(1.00) | 107(1.71) | 54(0.85) | 68(1.06) | 100(1.54) | 117(1.78) | 72(1.08) | 130(1.66) | 739 |
| Childcare patterns [n (%)] |  |  |  |  |  |  |  |  |  |  |
| Scattered children | 8788(65.60) | 9862(75.31) | 14826(66.05) | 8466(66.75) | 14303(67.08) | 11304(73.55) | 14664(72.85) | 8433(66.13) | 8301(68.13) | 98947(69.01) |
| Kindergarten children | 4148(30.96) | 2874(21.95) | 6771(30.17) | 3715(29.29) | 6200(29.08) | 3343(21.75) | 4480(22.26) | 3661(28.71) | 3300(27.08) | 38492(26.85) |
| Student | 440(3.28) | 319(2.44) | 776(3.46) | 460(3.63) | 767(3.60) | 655(4.26) | 896(4.45) | 605(4.74) | 481(3.95) | 5399(3.77) |
| Other patterns | 20(0.15) | 41(0.31) | 73(0.33) | 42(0.33) | 53(0.25) | 68(0.44) | 90(0.45) | 53(0.42) | 102(0.84) | 542(0.38) |
| Residence [n (10^5^)] |  |  |  |  |  |  |  |  |  |  |
| Yaohai | 2510(266.06) | 2313(246.06) | 3497(368.11) | 2039(212.4) | 3293(339.48) | 2265(231.29) | 3520(352.11) | 1933(190.27) | 2085(156.94) | 23455(258.12) |
| Luyang | 1018(163.45) | 1020(161.18) | 1710(265.94) | 1166(178.56) | 1882(284.29) | 1219(181.8) | 1575(230.77) | 738(106.62) | 864(123.91) | 11192(187.91) |
| Shushan | 2592(214.88) | 2686(224.51) | 4893(403.71) | 2933(239.43) | 5176(418.09) | 3710(296.42) | 4713(366.29) | 2989(227.77) | 2144(114.35) | 31836(269.72) |
| Baohe | 2345(267.85) | 1979(224.39) | 3356(375.39) | 2115(231.15) | 3456(372.01) | 2658(279.14) | 3349(345.29) | 2095(213.21) | 2218(182.18) | 23571(273.52) |
| Chaohu | 1283(167.09) | 1310(168.81) | 1824(233.25) | 1129(143.46) | 1897(240.74) | 1060(133.8) | 1347(169.18) | 964(120.02) | 814(111.94) | 11628(165.65) |
| Changfeng | 885(142.22) | 618(98.78) | 1161(183.99) | 601(93.91) | 1153(176.84) | 779(118.01) | 1460(218.89) | 730(108.28) | 1149(146.56) | 8536(143.31) |
| Feidong | 1097(129.89) | 986(115.4) | 1516(175.67) | 997(114.33) | 1681(191.02) | 1157(130.34) | 1351(150.75) | 1356(149.85) | 917(103.64) | 11058(140.2) |
| Feixi | 1281(173.33) | 1531(207.22) | 2104(282.04) | 1172(155.64) | 1626(214.23) | 1567(203.32) | 1702(218.15) | 1073(135.81) | 1537(158.86) | 13593(192.96) |
| Lujiang | 385(40.51) | 653(67.64) | 2385(244.62) | 531(53.91) | 1159(116.95) | 955(95.43) | 1113(110.35) | 874(86.23) | 456(51.34) | 8511(96.96) |
| Pathogen [n (%)] | 337(2.52) | 355(2.71) | 366(1.63) | 344(2.71) | 303(1.42) | 340(2.21) | 335(1.66) | 546(4.28) | 541(4.44) | 3467(2.42) |
| CV-A16 | 143(42.43) | 32(9.01) | 133(36.34) | 101(29.36) | 97(32.01) | 64(18.82) | 93(27.76) | 213(39.01) | 56(10.35) | 932(26.88) |
| EV-A71 | 134(39.76) | 185(52.11) | 161(43.99) | 57(16.57) | 66(21.78) | 74(21.76) | 2(0.60) | 1(0.18) | 3(0.55) | 683(19.70) |
| Other Enteroviruses ^a^ | 60(17.80) | 138(38.87) | 72(19.67) | 186(54.07) | 140(46.20) | 202(59.41) | 213(63.58) | 255(46.70) | 215(39.74) | 1481(42.72) |
| CV-A6 | - | - | - | - | - | - | 27(8.06) | 64(11.72) | 266(49.17) | 357(10.30) |
| CV-A10 | - | - | - | - | - | - | 0(0.00) | 13(2.38) | 1(0.18) | 14(0.40) |
| Severe cases [n (%)] | 11(0.08) | 22(0.17) | 70(0.31) | 3(0.02) | 2(0.01) | 3(0.02) | 3(0.01) | 0(0.00) | 0(0.00) | 114(0.08) |
| male | 9(81.82) | 13(59.09) | 47(67.14) | 1(33.33) | 1(50.00) | 1(33.33) | 2(66.67) | 0(0.00) | 0(0.00) | 74(64.91) |
| female | 2(18.18) | 9(40.91) | 23(32.86) | 2(66.67) | 1(50.00) | 2(66.67) | 1(33.33) | 0(0.00) | 0(0.00) | 40(35.09) |

^a^ Other Enteroviruses included CV-A6 and CV-A10 prior to 2018, but unincluded after 2018
